# Bcl2 is a critical regulator of bile acid homeostasis by dictating Shp and lncRNA H19 function

**DOI:** 10.1038/srep20559

**Published:** 2016-02-03

**Authors:** Yuxia Zhang, Chune Liu, Olivier Barbier, Rana Smalling, Hiroyuki Tsuchiya, Sangmin Lee, Don Delker, An Zou, Curt H. Hagedorn, Li Wang

**Affiliations:** 1 Department of Pharmacology, Toxicology & Therapeutics, University of Kansas Medical Center, Kansas City, KS 66160; 2 Department of Physiology and Neurobiology, and The Institute for Systems Genomics, University of Connecticut, Storrs, CT 062696; 3 Laboratory of Molecular Pharmacology, CHU-Québec Research Centre and Faculty of Pharmacy, Laval University, Québec, QC, Canada; 4 Department of Medicine, University of Utah School of Medicine, Salt Lake City, UT 84108; 5 Graduate School of Pharmaceutical Sciences, Osaka University, Japan; 6 Central Arkansas Veterans Healthcare System and University of Arkansas for Medical Sciences, Little Rock, AR; 7 Veterans Affairs Connecticut Healthcare System, West Haven, CT 06516; 8 Department of Internal Medicine, Section of Digestive Diseases, Yale University, New Haven, CT 06520.

## Abstract

Bile acid (BA) metabolism is tightly controlled by nuclear receptor signaling to coordinate regulation of BA synthetic enzymes and transporters. Here we reveal a molecular cascade consisting of the antiapoptotic protein BCL2, nuclear receptor Shp, and long non-coding RNA (lncRNA) H19 to maintain BA homeostasis. Bcl2 was overexpressed in liver of C57BL/6J mice using adenovirus mediated gene delivery for two weeks. Hepatic overexpression of Bcl2 caused drastic accumulation of serum BA and bilirubin levels and dysregulated BA synthetic enzymes and transporters. Bcl2 reactivation triggered severe liver injury, fibrosis and inflammation, which were accompanied by a significant induction of H19. Bcl2 induced rapid SHP protein degradation via the activation of caspase-8 pathway. The induction of H19 in Bcl2 overexpressed mice was contributed by a direct loss of Shp transcriptional repression. H19 knockdown or Shp re-expression largely rescued Bcl2-induced liver injury. Strikingly different than Shp, the expression of Bcl2 and H19 was hardly detectable in adult liver but was markedly increased in fibrotic/cirrhotic human and mouse liver. We demonstrated for the first time a detrimental effect of Bcl2 and H19 associated with cholestatic liver fibrosis and an indispensable role of Shp to maintain normal liver function.

Bile acids (BAs), synthesized from cholesterol in the liver, play a critical role in eliminating excess cholesterol from the body and facilitating intestinal digestion and absorption of dietary fat, steroids, drugs, and lipophilic vitamins^1^. Extensive research has revealed bile acids as important signaling molecules in the regulation of lipid, glucose, and energy metabolism. However, excessive accumulation of cytotoxic bile acids can cause cell damage leading to inflammation and fibrosis contributing to carcinogenesis in gastrointestinal tract^2^. The nuclear receptor small heterodimer partner (Shp, Nr0b2) is an essential component in the negative feedback regulation of bile acid synthesis. In response to elevated levels of intrahepatocellular bile acids, nuclear receptor farnesoid X receptor (FXR) activates Shp to repress the expression of two key enzymes cholesterol 7α-hydroxylase (Cyp7A1) and sterol 12α-hydroxylase (Cyp8B1) for *de novo* synthesis of bile acids^3^,^4^,^5^. Additionally, induction of Shp represses the transactivation of bile acid transporter sodium-taurocholate cotransporting polypeptide (NTCP) at the basolateral membrane of the hepatocytes to block uptake of bile acids into hepatocytes^6^ and facilitates renal excretion of bile acids. In the intestine, increased bile acids activate FXR causing induction of fibroblast growth factor 15 (Fgf15, FGF19 in human), an intestinal hormone that binds with liver FGF receptor 4 (FGFR4) via an endocrine mode after its secretion from the intestine, to inhibit Cyp7A1^7^. Thus, bile acid homeostasis is tightly controlled by a coordinated regulation of genes involved in bile acid biosynthesis, uptake, and efflux in the liver and ileum.

Apoptosis is essential for maintaining tissue homeostasis. B-cell lymphoma protein 2 (Bcl2), an antiapoptotic family member containing four conserved α-helical motifs known as Bcl-2 homology (BH1–4) domains and a transmembrane domain (TM), is known to inhibit apoptosis by binding to the pro-apoptotic proteins BCL2-associated X protein (Bax) and BCL2-antagonist/killer 1 (Bak)^8^. Bcl2 expression is barely detectable in normal hepatocytes. However, its expression is highly induced in a rat model of cholestasis by bile-duct ligation (BDL)^9^ and in bile ducts and hepatocytes in human hepatitis C cirrhotic liver^10^. In addition, biliary epithelial cells are Bcl2 positive in primary biliary cirrhosis (PBC), an inflammatory condition of bile ducts that leads to fibrosis and cirrhosis^11^. Bcl2 expression in proliferated bile duct epithelial cells is proposed to promote hepatic stellate cell activation and fibrosis in patients with autoimmune cholangitis and PBC^12^. Interestingly, ursodeoxycholic acid (UDCA), a secondary bile acid (BA) produced by intestinal bacteria as a metabolic by-product, is effective in inducing tumor cells apoptosis via changing the conformation of Bcl2^13^. Despite these observations, the role of Bcl2 in BA metabolism and normal liver function remains unknown.

The long non-coding RNA (lncRNA) H19 is an imprinted and maternally expressed gene, which was first discovered in fetal mouse and human liver^14^. H19 is important in embryonic development and stem cell growth and its hepatic expression is repressed after birth^15^. In liver and gastric cancers, aberrant expression of H19 is observed^16^. Nonetheless, little is known about the regulatory role of H19 in liver disease.

This study describes a molecular cascade consisting of sequential translational and transcriptional regulatory events that coordinately maintain BA homeostasis, including Bcl2, Shp, and lncRNA H19.

## Results

### Hepatic overexpression of Bcl2 disrupts bile acid homeostasis

**Figure 1:**
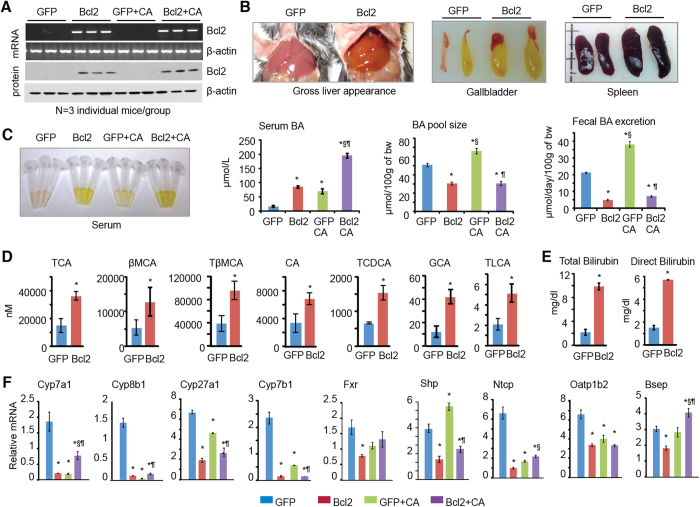
Hepatic overexpression of Bcl2 disrupts bile acid (BA) homeostasis. (**A**) qPCR and Western blot of liver Bcl2 mRNA and protein, respectively. Bcl2 was expressed in C57BL6 mice by adenovirus mediated gene delivery for two weeks. A second group of mice received 1% cholic acid (CA) feeding for 7 days one week post adenovirus administration. (**B**) Gross morphology of liver, gallbladder, and spleen. (**C**) Serum from each mouse group (*left*) and measurement of serum BA, BA pool size, and fecal BA excretion (*right*). (**D**) BA composition was analyzed using liquid chromatography-tandem mass spectrometry (LC-MS/MS). (**E**) Measurement of serum total bilirubin and direct bilirubin. (**F**) qPCR of genes involved in BA metabolism. Data are represented as mean ± SD in all panels except (**A,B**) *P < 0.05 *vs.* GFP, ^§^*P* < 0.05 *vs.* Bcl2, ^¶^*P* < 0.05 *vs.* GFP CA.

To examine the *in vivo* pathophysiological function of Bcl2, we transiently overexpressed Bcl2 specifically in the liver using adenovirus (control GFP and GFP-Bcl2) mediated gene delivery for two weeks. A second group of mice were fed a 1% cholic acid (CA) diet for 7 days (serve as a positive control) one week after virus injection. RT-PCR (Fig. 1A*, top two*) and Western blot (bottom two) analysis confirmed the expression of Bcl2 mRNA and BCL2 protein, respectively. Bcl2 levels were compatible between Bcl2 vs. Bcl2-CA groups, suggesting that they were not altered by CA feeding. Bcl2 overexpression caused a yellowish color in liver, an enlarged size of gallbladder and spleen (Fig. 1B). A yellowish color was also observed in serum in Bcl2 mice (Fig. 1C, *left*). Bcl2 markedly elevated serum bile acid levels, which were compatible with that of CA feeding. Serum BA levels were further increased in Bcl2+CA mice compared to Bcl2 mice. Interestingly, BA pool size and fecal BA secretion were both reduced in Bcl2 mice, which was in contrast to their elevation in CA-fed mice (Fig. 1C, *right two*). Therefore, the decreased secretion of BA through intestine to feces in Bcl2 mice will cause BA retention in gallbladder and result in an enlarged gallbladder.

To understand Bcl2-mediated BA metabolic changes, we determined alterations of BA composition in GFP and Bcl2 mice. Unconjugated, taurine-, glycine- and sulfate-conjugated bile acid levels were measured using liquid chromatography/tandem mass spectrometry (LC-MS/MS). Among 19 BAs we analyzed, taurocholic acid (TCA), β-muricholic acid (βMCA), and TβMCA, the three major BAs in mouse serum, were increased significantly in Bcl2 mice (Fig. 1D). The primary bile acid CA and other conjugated BAs such as taurochenodeoxycholic acid (TCDCA), glycocholic acid (GCA), and taurolithocholic acid (TLCA) were elevated as well. Significant increases in total bilirubin and direct bilirubin, two indicators of impaired liver function, were observed in Bcl2 mouse serum (Fig. 1E).

BA synthesis and absorption are systematically controlled by many genes, including hepatic BA synthetic enzymes, nuclear receptors, and BA transporters. Interestingly, the expression of key enzymes for BA synthesis (Cyp7a1, Cyp8b1, Cyp27a1, Cyp7b1), BA receptor Fxr and its downstream target Shp, and several BA transporters (Ntcp, Oatp1b2, Bsep), was consistently downregulated in Bcl2 vs. GFP liver (Fig. 1F). The results suggest a pervasive disruption of BA homeostasis by Bcl2 reactivation in the liver.

### Bcl2 reactivation induces liver injury, fibrosis, and H19 expression

**Figure 2:**
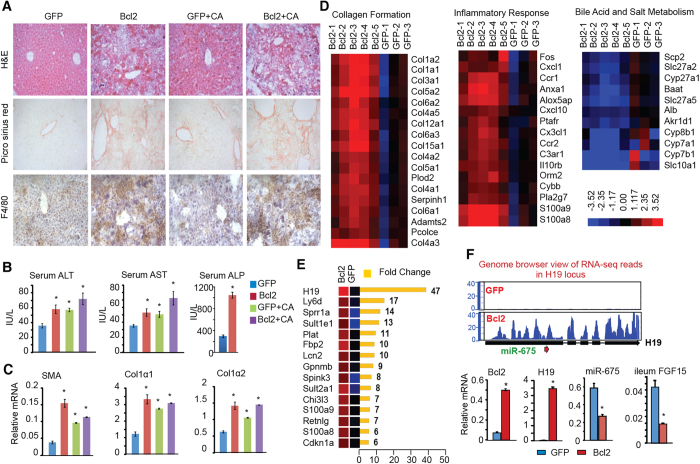
Hepatic overexpression of Bcl2 results in cholestatic liver fibrosis and up-regulates H19. (**A**) H&E staining, picrosirius red staining of collagen, and IHC staining of macrophage marker F4/80 in liver sections. Representative staining examples are shown at a magnification of 20X. (**B**) Measurement of serum alanine aminotransferase (ALT), aspartate aminotransferase (AST), and alkaline phosphatase (ALP) levels. (**C**) qPCR of liver fibrogenic genes alpha smooth muscle actin (α-SMA), collagen type I alpha 1 (Col1a1), and collagen type I alpha 2 (Col1a2). (**D**) Heat map from RNA-seq results showing differentially expressed genes (DEG) involved in collagen formation (*left*), inflammatory response (*middle*), and bile salt metabolism (*right*). (**E**) Top ten greatest induced genes in Bcl2 mice. (**F**) *Top*: Genome browser view of RNA-seq reads in H19 locus. *Bottom*: qPCR of liver Bcl2, H19, miR-675, and ileum Fgf15 expression in GFP or Bcl2 mice. Data are represented as mean ± SD in (**B,C,F**) *P < 0.05 Bcl2 *vs.* GFP.

H&E staining revealed severe liver damage with changes in both liver architecture as well as necrosis in Bcl2 mice (Fig. 2A and S1). The development of liver fibrosis and Kupffer cells infiltration was identified by picrosirius red and F4/80 staining, respectively. Interestingly, the increases in serum alanine aminotransferase (ALT) and aspartate aminotransferase (AST) levels, two classical markers of liver injury, were less striking than we would expect in Bcl2 mice (Fig. 2B, left and middle), whereas serum alkaline phosphatase (ALP) levels were drastically increased in Bcl2 mice (right). This observation is similar to that in patients with HCV cirrhosis who often have normal or slightly elevated serum AST and ALT levels^17^. Consistent with the histology analysis, the expressions of fibrosis markers, including alpha smooth muscle actin (α-SMA), collagen type I alpha 1 (Col1a1), and collagen type I alpha 2 (Col1a2), was significantly upregulated in Bcl2 vs GFP liver (Fig. 2C). Adenovirus-mediated gene delivery is one of the most commonly used methods to effectively deliver gene of interests in the liver. The observed liver injury by Ad-BCL2 was unlikely attributed by the mild inflammation by adenovirus, as it was not observed with the AdGFP-control.

A revolution in the analysis of RNA has come through the development of deep sequencing (RNA-seq) technologies to map the entire transcriptome in cells. To identify crucial regulators contributing to Bcl2-mediated liver injury, we conducted RNA-seq analysis of liver in Bcl2 (n = 5) and GFP control (n = 3) mice (GEO number: GSE67972). A total of 2164 differentially expressed genes (DEG) were identified (fold change >1.50 and false discovery rate (FDR) <0.05) in Bcl2 compared to GFP liver. Unsupervised hierarchical clustering of the log-transformed gene expression reads for each sample produced a visual Bcl2 overexpression transcriptome signature of 1091 upregulated and 1073 downregulated genes (Fig. S2A). As expected, the expression of multiple genes involved in collagen formation (Fig. 2D*, left*), inflammation (Fig. 2D*, middle*) and lipid metabolism (Fig. S2B) was upregulated in Bcl2 mice. In contrast, genes involved in BA synthesis were downregulated (Fig. 2D*, right*), consistent with the qPCR results (Fig. 1F). We used Gene Set Enrichment Analysis (GSEA) computational method and Ingenuity Pathway Analysis (IPA) software to identify canonical pathways and biological processes that were enriched in the Bcl2 DEG set. Multiple pathways were significantly altered in Bcl2 liver (Fig. S3).

The most striking observation was that hepatic H19 expression was induced 47 fold in Bcl2 compared to GFP mice (Fig. 2E). The induction of H19 was visualized in the integrated genome browser view of the H19 locus (Fig. 2F*, top*) and verified by qPCR (*bottom*). The miR-675 was encoded in H19 exon1 and was reportedly co-activated with H19 during muscle differentiation^18^. However, miR-675 is barely detectable in fetal liver despite the vast levels of H19, suggesting that the expression of miR-675 and H19 is not co-regulated in liver^19^. Insulin-like factor 2 (Igf2) and H19 genes lie 70-kb apart on chromosome 7 and are reciprocally imprinted^19^. In contrast to H19, Igf2 expression was minimally altered in Bcl2 mice (not shown), suggesting a specific reactivation of H19 in Bcl2 mice. The ileum Fgf15 mRNA was markedly diminished in BCL2 mice (Fig. 2F, right). Therefore, H19 reactivation was likely associated with Bcl2-induced liver injury.

### Bcl2 destabilizes SHP protein which involves the activation of caspase 8 pathway

**Figure 3:**
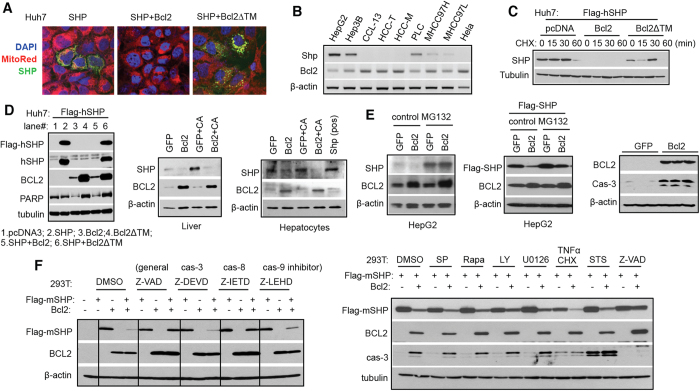
Bcl2-mediated SHP protein degradation is associated with activation of caspase 8 pathway. (**A**) Micrographs of DAPI staining (blue), MitoTracker staining (red), and GFP-SHP expression (green) in Huh7 cells transfected with GFP-SHP plasmid, alone or in combination with wide-type Bcl2 (Bcl2wt) or Bcl2 lacking the TM domain (Bcl2ΔTM). Magnification of 20X. (**B**) RT-PCR of Shp and Bcl2 mRNAs in various cells. (**C**) Western blot of SHP protein in Huh7 cells that were expressed with Bcl2 or Bcl2ΔTM in the absence or presence of protein synthesis inhibitor cycloheximide (CHX). (**D**) *Left:* Western blot of SHP and BCL2 proteins in Huh7 cells. SHP protein was detected by anti-Flag or anti-SHP antibody. *Middle:* Western blot of SHP and BCL2 proteins in the liver. The mice were the same as in Fig. 1. *Left:* Western blot of SHP and BCL2 proteins in primary mouse hepatocytes. The corresponding mRNA level is in Fig. S4A. Hepatocytes infected with ade-SHP served as a positive control. (**E**) *Left and middle:* Western blot of SHP and BCL2 proteins in HepG2 cells treated with DMSO control or 5 μM MG132 for 6 hr. The endogenous SHP protein and exogenously expressed Flag-SHP protein were detected by anti-SHP or anti-Flag antibody, respectively. *Right*: Western blot of BCL2 and cas-3 proteins in Bcl2 overexpressed liver. (**F**) Western blot of SHP and BCL2 proteins in HEK 293T cells. *Left:* Cells were pre-treated with caspase inhibitors (50 μM) for 1 hr followed by plasmid transfection for 24 hr. *Right:* Cells were pre-treated with various inhibitors for 1 hr followed by plasmid transfection for 24 hr. For TNFα+ CHX and staurosporine groups, cells were transfected with plasmids for 20 hr followed by a 4 hr treatment. Dose: SP600125 (SP) 50 μM, Rapamycin (Rapa) 10 μM, LY294002 (LY) 50 μM, U0126 10 μM, TNFα 50** **ng/ml + CHX 50 μM, staurosporine (STS) 1 μM, and Z-VAD 50 μM.

We recently showed that SHP interacts with BCL2 protein via BCL2 TM domain *in vitro*^20^. Micrograph analysis revealed that SHP was expressed and localized in the cytoplasmic compartment in Huh7 cells and that ectopic expression of Bcl2, but not Bcl2 without a TM domain (Bcl2∆TM), resulted in a complete loss of SHP protein (Fig. 3A). Interestingly, the expression of Shp exhibited a negative correlation with Bcl2 in various cells (Fig. 3B). The downregulation of SHP by Bcl2 occurred in the presence of protein synthesis inhibitor cycloheximide (CHX) (Fig. 3C), suggesting that the reduced SHP protein was due to its increased degradation. In contrast to the effect of Bcl2 on SHP, BCL2 protein levels were not altered by SHP co-expression (Fig. 3D, *left*). As expected, the endogenous SHP protein induced by CA was also markedly reduced by ectopically expressed Bcl2 in mouse liver and primary hepatocytes (Fig. 3D, *middle and right*). In contrast, Shp mRNA was not decreased by Bcl2 overexpression (Fig. S4A). The rapid degradation of SHP protein by Bcl2 appeared to be a common rather than cell type specific phenomenon, as it occurred in a variety of cell types, including Huh7, HepG2, Hela, Hepa1, HT29, and 293T (Fig. 3 and S4B-E).

SHP protein was shown to undergo proteasome degradation^21^, which is mediated by Mdm2 E3 ligase^22^. However, proteasome inhibitor MG132 only partially blocked (Fig. 3E, *left two*) or failed to block SHP protein degradation by Bcl2 (Fig. S5A), depending on the cell type. Overexpression of Bcl2 caused a significant caspase 3 cleavage, suggesting that Bcl2-induced liver injury was mediated by caspase activation (Fig. 3E, *right*). In mammalian cells, two major pathways (extrinsic and intrinsic) leading to caspases activation have been described involving caspase 8 (cas-8), caspase 9 (cas-9) and caspase 3 (cas-3) (Fig. S5B). General caspase inhibitor Z-VAD and cas-8 inhibitor Z-IETD, but not cas-3 inhibitor Z-DEVD and cas-9 inhibitor Z-LEHD, completely stabilized SHP protein in the presence of Bcl2 (Fig. 3F*, left*), suggesting that cas-8 activation is functionally involved in SHP protein destabilization by Bcl2.

To define the detailed molecular basis, we examined potential contributions of different signaling pathways in Bcl2-mediated SHP protein degradation. We treated 293T cells with JNK inhibitor SP600125 (SP), mTORC1 inhibitor Rapamycin (Rapa), PI3 Kinase inhibitor LY294002 (LY), MEK1/2 inhibitor U0126, cas-8 activator TNFα and Cycloheximide (CHX), intrinsic apoptosis inducer staurosporine (STS), as well as general caspase inhibitor Z-VAD. TNFα and CHX, which are known to activate cas-8 pathway, triggered SHP protein autodegradation and enhanced SHP degradation by Bcl2 (Fig. 3F*, right*). In the presence of Z-VAD, but not other signaling pathway inhibitors, SHP protein degradation by Bcl2 was essentially impeded. This was accompanied by a concomitant inactivation of cas-3 and its cleavage. The results demonstrate that Bcl2 induces caspase 8 activation leading to rapid SHP protein degradation.

### H19 expression is transcriptionally repressed by Shp

**Figure 4:**
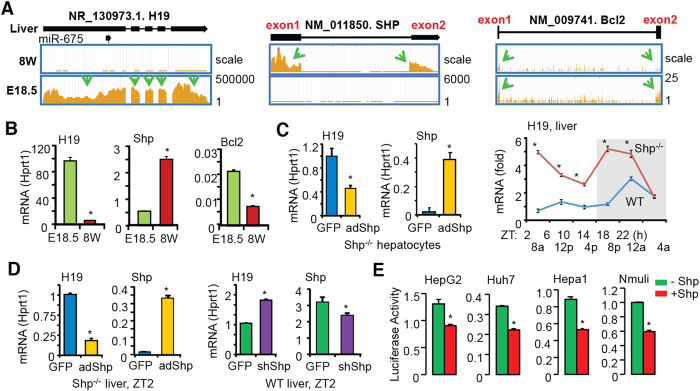
Shp is a transcriptional repressor of H19 expression. (**A**) Integrated genome browser visualization of RNA-Seq read coverage for liver H19, SHP, and Bcl2 in WT mice at E18.5 and 8 wk. (**B**) qPCR of H19, Shp, and Bcl2 mRNAs. *P < 0.05, 8 wk *vs.* E18.5. (**C**) *Left:* qPCR of H19 expression in *Shp*^−/−^ hepatocyte with *Shp* re-expression. *P < 0.05 *Shp vs.* GFP. *Right:* qPCR of hepatic H19 expression of WT and *Shp*^**−/−**^ mice. Livers were collected over a 12** **h/12** **h light/dark cycle. N = 5 mice/time point. *P < 0.05 *Shp*^**−/−**^ *vs.* WT. (**D**) qPCR of H19 expression in *Shp*^−/−^ liver with *Shp* re-expression or in WT liver with *Shp* knockdown. N = 5 mice/group. *P < 0.05 *vs.* GFP. (**E**) Luciferase reporter assay in hepatic cells transfected with *H19*-Luc and expression plasmid for *Shp*. *P < 0.05 *vs.* respective control.

RNA-seq (Fig. 4A) and qPCR (Fig. 4B) analysis revealed an inverse correlation between H19 and Shp at E18.5 (high H19, low Shp) and 8W (low H19, high Shp) of mouse liver. Based on our knowledge that Shp generally functions as a transcriptional repressor^4^, we postulated that H19 gene transcription might be repressed by Shp in adult liver. Re-expression of Shp in *Shp*^−/−^ hepatocytes inhibited H19 mRNA (Fig. 4C*, left*). Shp expression is under the control of liver circadian clock^23^, therefore we examined H19 expression in WT and *Shp*^−/−^ liver collected over a 12 h/12 h light/dark cycle. Indeed, H19 mRNA exhibited a circadian rhythmic expression in WT mice, which was notably increased in *Shp*^−/−^ mice (Fig. 4C*, right*). In addition, re-expression of Shp in *Shp*^−/−^ liver (Fig. 4D*, left two*) inhibited H19 mRNA, whereas knockdown of Shp in WT liver induced H19 mRNA (Fig. 4D*, right two*). These results provide direct *in vivo* evidence for a transcriptional repression of H19 by Shp.

We cloned the minimal promoter of H19 into a luciferase reporter (Fig. S6) based on its published genomic structure^24^. Transient transfection assays showed that Shp markedly repressed H19 luciferase reporter activity (Fig. 4E). Such regulation occurred in human (HepG2, Huh7) and mouse (Hepa1, Nmuli) hepatocyte derived cells, suggesting a common regulatory mechanism governing H19 expression by Shp in both mouse and human liver. Thus, we identified Shp as a new repressor of H19 gene transcription.

### Bcl2-induced liver injury is alleviated by knockdown of H19 or re-expression of Shp

**Figure 5:**
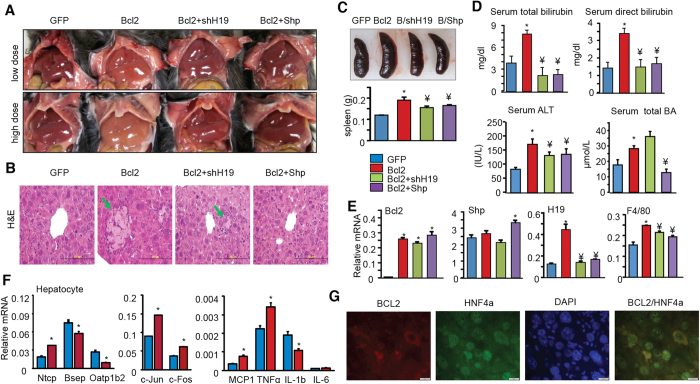
H19 knockdown or Shp re-expressing alleviates liver injury induced by Bcl2. (**A**) Gross morphology of liver. Low dose: 1** × **10^10^ viral particles/ml; high dose: 3** × **10^10 ^viral particles/ml. (**B**) H&E staining in liver sections. A representative image from low dose treatment group is shown. Arrows indicate regions of coagulated hepatocyte necrosis. Scale bar: 200 μm. (**C**) Gross morphology of spleen (*top*) and the measurement of spleen weight (*bottom*). (**D**) Serum measurements of total or direct bilirubin, ALT, and total BA in low dose groups. (**E**) qPCR of liver Bcl2, Shp, H19, F4/80 expression in low dose groups. Data are represented as mean ± SD in all panels except (**A,B**) *P < 0.05 *vs.* GFP; ^¥^*P* < 0.05 *vs.* Bcl2. (**F**) qPCR of gene expression in primary hepatocytes transduced with Bcl2. *P < 0.01 *vs.* GFP. (**G**) Immunohistochemistry of BCL2 and HNF4α protein localization in Bcl2 overexpressed mouse liver.

To dissect out the contribution of H19 induction or decrease in SHP protein to Bcl2-induced liver injury, we knocked-down H19 and re-expressed Shp in Bcl2 livers respectively. We first established that the effect of low dose Bcl2 virus (1 × 10^10^ viral particles/ml) was similar to that of high dose (3 × 10^10^ viral particles/ml); both induced a yellowish liver color (Fig. 5A). To better observe a rescuing effect of shH19 or Shp, a low dose Bcl2 virus was introduced into mouse liver and the samples from the low dose groups were used for subsequent analysis.

Bcl2 overexpression induced liver necrosis and inflammation (Fig. 5B), spleen enlargement (Fig. 5C), increased serum bilirubin, ALT and BA levels (Fig. 5D). Most of the abnormal serum parameters in Bcl2 mice were corrected by shH19, except that liver necrosis was reduced but not prevented (Fig. 5B), and serum BA levels were slightly increased. Although being only moderately increased in Bcl2 mice (Fig. 5E*, 2*^*nd*^ *panel*), re-expression of Shp fully rescued mice from developing liver necrosis. In addition, the increased levels of serum bilirubin, ALT and BA by Bcl2 were decreased to almost basal levels by Shp re-expression (Fig. 5D,E). It was noted that shH19 and Shp only partially alleviated liver inflammation as evidenced by the diminished F4/80 mRNA in Bcl2+shH19 and Bcl2+SHP mice vs Bcl2 mice (Fig. 5E, *4*^*th*^ *panel*). H19 knockdown did not affect the reduction of Cyp7a1, Cyp8b1 and Fxr in Bcl2 mice, whereas Shp re-expression alleviated the repression of Cyp7a1 and Fxr by Bcl2 (Fig. S5). As expected, H19 levels were markedly reduced in Bcl2+Shp compared to Bcl2 mice (Fig. 5E*, 3*^*rd*^ *panel*), which was consistent with a repression by Shp (Fig. 4). Furthermore, BCL2 overexpression in primary hepatocytes activated bile acid transporter Ntcp but repressed Bsep and Oatp1b2 (Fig. 5F). BCL2 also induced c-Jun, c-Fos and several inflammatory genes. We used HNF4α as a marker, which is expressed in the nucleus of hepatocytes. BCL2 is largely expressed in the cytoplasm of hepatocytes (Fig. 5G). The results from our rescuing experiments demonstrated that the disrupted liver metabolic homeostasis by Bcl2 was contributed by both the diminished Shp and enhanced H19 expression and function.

### Bcl2 and H19 are highly induced in fibrotic and cirrhotic human and mouse liver

**Figure 6:**
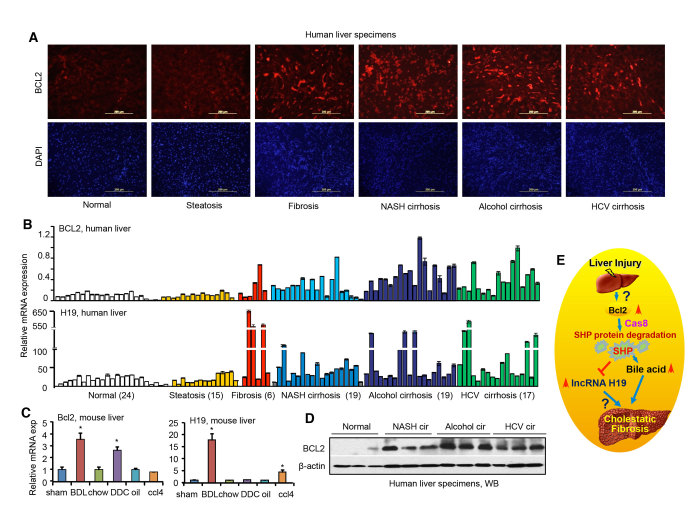
The expression of Bcl2 and lncRNA H19 is induced in fibrotic and cirrhotic liver. (**A**) Immunohistochemistry of BCL2 protein in human liver specimens. Representative staining examples from each group are shown. Scale bar: 200 μm. (**B**) qPCR of BCL2 and H19 expression in human liver specimens. (**C**) qPCR of Bcl2 and H19 expression in mouse models of liver fibrosis, including bile duct ligation (BDL), 3,5-diethoxycarbonyl-1,4-dihydrocollidine (DDC), or carbon tetrachloride (CCl4) (n = 5/group). Data are represented as mean ± SD. *P < 0.05, BDL, CCl4, or DDC *vs.* respective control. (**D**) Western blots (WB) of BCL2 protein in human normal, NASH cirrhotic, alcohol cirrhotic, and HCV cirrhotic liver specimens (n = 3/group). (**E**) Schematic of Bcl2/Shp/lncRNA H19 circuit to modulate BA homeostasis and cholestatic liver fibrosis. The basal Bcl2 expression in normal liver is low, which is induced in fibrotic/cirrhotic liver by mechanisms that are currently unknown. Overexpression of Bcl2 or its reactivation by 26 liver injury causes a rapid SHP protein degradation that involves Cas8 activation, which subsequently results in disruption of BA homeostasis and cholestatic liver fibrosis. Consequently, H19 expression is induced by loss of Shp transcriptional repression. The role of H19 in the development of liver fibrosis remains to be elucidated in future studies.

To assess the clinical relevance of BCL2 and H19 in chronic liver diseases, we examined their expression in human liver specimens. Immunohistochemistry (IHC) staining revealed a strong induction of BCL2 protein in liver fibrosis, NASH, alcohol and HCV cirrhosis relative to normal livers (Fig. 6A). qPCR revealed a marked induction of BCL2 mRNA and a more substantial and differential induction of H19 mRNA in human fibrotic and cirrhotic liver specimens (Fig. 6B), regardless of the underlying disease.

Hepatic Bcl2 and H19 expression was further analyzed in several mouse models of liver fibrosis, including two cholestatic models, bile-duct ligation (BDL), 3,5-diethoxycarbonyl-1,4-dihydrocollidine (DDC), and one toxic chemical model, carbon tetrachloride (CCl4)^25^. Liver Bcl2 mRNA was significantly induced by BDL and DDC, whereas H19 expression was markedly induced by BDL and to a lesser extent by CCl4 (Fig. 6C). We further confirmed the elevation of BCL2 protein levels in nonalcoholic steatohepatitis (NASH), alcohol and HCV cirrhosis by Western blot (Fig. 6D).These results demonstrated a positive correlation of activation of BCL2 and H19 with the development of liver fibrosis and cirrhosis in both humans and mice.

## Discussion

Much progress has been made in characterizing the enzymatic pathways of bile acid and cholesterol synthesis and in understanding how these pathways are regulated by nuclear receptors. Relatively recent major breakthroughs have included the discovery of the role of G-protein-coupled receptor (GPCR)^26^ and fibroblast growth factor (FGF) signaling^7^ in bile acid metabolism. Each of these has been pursued vigorously as potential therapeutic targets^27^.

A major finding of our study is the identification of Bcl2 as a new regulator of bile acid homeostasis. Bcl2 gene was originally identified at a breakpoint of translocations commonly occurring in follicular B cell lymphomas and was later found to play a key role in cell survival and inhibition of apoptosis^8^. The basal Bcl2 expression is extremely low in normal liver, which raises the question whether Bcl2 has a physiological function in the liver. In this study, we present convincing evidence that provides an explanation why low Bcl2 levels are essential to maintain normal hepatic homeostasis.

We show that forced expression of Bcl2 in liver disrupts BA homeostasis leading to severe cholestatic liver fibrosis, which is accompanied by the drastic induction of H19. At the molecular level, we show that overexpression of Bcl2 causes a rapid SHP protein degradation contributing to the dysregulation of BA metabolism. In addition, we identify SHP as a novel transcriptional repressor of H19 expression. SHP protein degradation by Bcl2 results in the loss of Shp inhibition of H19 contributing to H19 induction in Bcl2 mice. The replenishment of Shp reverses the phenotype seen in Bcl2 mice and significantly improves liver function and that knockdown H19 by shH19 partially prevents liver injury by Bcl2. Taken as a whole, the present study uncovers Bcl2/Shp/H19 molecular cascade as a new regulatory component of BA metabolism (Fig. 6D). It is clear that reactivation of Bcl2 has a detrimental effect on normal liver function. On the other hand, Bcl2 expression is highly induced in fibrotic and cirrhotic livers. It is likely that liver injury causes Bcl2 upregulation and the induction of Bcl2 further facilitates liver damage and disease progression at least in part through diminishing Shp function. This study is potentially significant because it may allow us to explore BCl2 as a promising drug target for liver fibrosis and cirrhosis.

Many papers have investigated the effects of bcl2 overexpression in mouse liver by adenoviral delivery or transgenic approach^28^,^29^. For example, in Rodriguez’s study^28^, BCL2 was moderately expressed in transgenic mice. Our study shows that the severity of liver injury is associated with the level of BCL2 (high dose and more severe in Fig. 2A vs low dose in Fig. 5B). In Bilbao’s study^29^, a BCL2 mutant without the transmembrane domain (TM) was delivered by adenovirus. We show that the TM domain of BCL2 is critical for BCl2 and SHP interaction and consequently the SHP protein degradation by BCL2. Therefore, it is unexpected that BCL2-without-TM will not cause liver injury, which is consistent with Bilbao’s observation.

Shp is a transcriptional repressor of Cyp7A1 and Cyp8B1 expression and the loss of Shp is expected to increase BA synthesis and an overall increase in BA pool size, serum BA levels and BA excretion^5^. It is noted that in Bcl2 overexpressed mice with SHP protein degradation, serum BA is highly elevated, whereas BA pool size and fecal BA levels are reduced. BAs are known to induce inflammatory cytokines to activate the MAPK/JNK signaling pathway that inhibits CYP7A1 gene transcription^30^. It is anticipated that the severe liver inflammation and injury caused by Bcl2 not only disrupts BA biosynthesis, but also BA transport. Indeed, the entire BA metabolic program is noticeably interrupted in Bcl2 overexpressed mice. In addition, a non-SHP-dependent mechanism may also be involved in BA dysregulation in response to Bcl2 overexpression. It should be noted that the gut Fgf15 signaling plays a critical role in repressing liver BA synthesis^7^. Due to the lack of reliable commercial kit to measure serum Fgf15 levels in mice, we were unable to accurately examine Fgf15 levels in Bcl2 mice. One of our future focuses would be to establish the method developed by the Kliewer/Mangelsdorf group^31^ that would allow us to determine the role of Fgf15 in Bcl2 mediated BA metabolism.

The expression of H19 was increased in liver cancer^32^. H19 was reported to either promote^33^ or inhibit^34^ cancer cell invasion depending on the cell type. Our study is the first to reveal a role of H19 in cholestatic liver disease. A recent report described an induction of H19 in the cirrhotic CCl4 model^35^, which is in agreement with our observation of a significant induction of H19 in human fibrotic/cirrhotic liver and by BDL and CCl4 in mouse models. The molecular basis by which H19 regulates the development of liver fibrosis remains to be elucidated using H19 knockout mice in future studies.

In summary, our study reveals a novel molecular cascade involving Bcl2, Shp and H19 to keep the normal liver function in check. Further studies are necessary to dissect out cell type specific roles of Bcl2 and H19 in liver fibrosis and other chronic liver diseases.

## Materials and Methods

### Cell Lines

Human hepatoma cell line Huh7 (Health Science Research Resources Bank JCRB0403), HepG2 (ATCC HB-8065), mouse hepatoma cell line Hepa1 (ATCC CRL-1830), human embryonic kidney 293T cells (ATCC CRL-3216), human cervical carcinoma cell line Hela (ATCC CCL-2), and human colorectal carcinoma cell line HT29 (ATCC HTB-38) were maintained in Dulbecco’s modified Eagle’s medium (DMEM) with 100 U of penicillin G-streptomycin sulfate/ml and 10% heat-inactivated fetal bovine serum (FBS).

### Chemicals, Plasmids, adenoviruses, and antibodies

Chemicals including cholic acid (CA), 3,5-diethoxycarbonyl-1,4-dihydrocollidine (DDC), carbon tetrachloride (CCl4), and collagenase IV were purchased form Sigma. The mouse H19 promoter luciferase reporter (H19-Luc), expression plasmids for Flag-mouse SHP, Flag-human SHP, and GFP-human SHP were cloned in our laboratory and confirmed by sequencing. Expression plasmids for Bcl2 full length (Bcl2 wt) and Bcl2 mutant without a TM domain (Bcl2ΔTM) were provided by Dr. Xiaokun Zhang (Sanford Burnham Institute for Medical Research). Adenoviruses for GFP, Shp, shSHP were described previously^36^. Adenoviruses with the validated sequence for knock down mouse H19 (GCATGACAGACAGAACATT) was generated by Welgen (Worcester, MA). The following antibodies were used for protein precipitation (IP) and Western blots (WB): antibodies against Flag (Sigma, F7425), GFP (Sigma, G1544), Bcl2 (Abcam, ab7973), β-actin (Sigma, A-1978), α-Tubulin (Sigma, T6074), PARP (Cell signaling, #9542), SHP (Santa Cruz, sc-30169), caspase-3 (Cell signaling, #9661), cleaved caspase-8 (Cell signaling, #9748).

### Human Liver Specimens

The coded human liver specimens were obtained through the Liver Tissue Procurement and Distribution System (Minneapolis, Minnesota) and have been described previously^25^. Because we don’t ascertain individual identities associated with the samples, the Institutional Review Board for human research committee at University of Utah determined that the project is not research involving human subjects.

### Animal Treatment

C57BL/6J mice were purchased from Jackson Laboratory (Bar Harbor, ME, USA). *Shp*^**−/−**^ (SKO) on C57BL/6J background was described previously^25^. Mice were fed a standard rodent chow (Harlan No. 2020X) with free access to water and maintained in a 12 hr light/dark (LD) cycle (light on 6 AM to 6 PM), temperature-controlled (23 °C), and virus-free facility. Experiments on mice were performed on males at the age of 8 weeks unless stated otherwise (n = 5/group). The treatments of mice with 3,5-diethoxycarbonyl-1,4-dihydrocollidine (DDC) supplemented diet, bile duct ligation (BDL), and carbon tetrachloride (CCl4) have been described previously^25^. For *in vivo* adenoviral transduction, mice were injected via tail vein with purified adenoviruses at 3** × **10^10^ (high dose) or 1** × **10^10^ (low dose) virus particles per mouse. All experiments were performed in accordance with relevant guidelines and regulations approved by the Institutional Animal Care and Use Committee (ICAUC) at the University of Utah and University of Connecticut.

### Histological analysis of liver sections

Fresh liver tissues were either fixed with formalin for paraffin section or immediately embedded in O.C.T. compound for frozen section. For hematoxylin and eosin (H&E) staining, Masson’s trichrome staining, and picrosirius red staining, paraffin sections at thickness of 4 μm were cut and subjected to xylene and ethanol rehydration prior to staining. Frozen sections were cut at 10 μm for immunohistochemistry staining.

### Western Blots

Cells overexpressed with various plasmids were lysed in lysis buffer (50 mm Tris, pH 8.0, 1% Nonidet P-40, 150 mm NaCl, 0.5% sodium deoxycholate, 0.1% SDS) with protease inhibitors (Thermo Fisher Scientific, #78410). Cell lysates (30 μg) were resolved by SDS-PAGE and transferred to nitrocellulose membranes according to standard procedures. Membranes were blocked, incubated with primary antibodies followed by horseradish peroxidase-conjugated corresponding secondary antibody incubation. Antibody binding was visualized with SuperSignal West Pico Chemiluminescent Substrate (Thermo Fisher Scientific, #34080) according to the manufacturer’s protocol. Equal loading of protein was verified with β-actin or α-Tubulin.

### Transient transfection and Promoter activity assays

Cells seeding in 24 well plates were transfected with mouse H19-Luc reporter and indicated expression plasmids in the figure legends by Lipofectamine 2000 (Invitrogen). Luciferase and renilla activities were determined by luciferase and renilla assay systems (Promega, Madison, WI) at 48 hr post-transfection. Luciferase activities were normalized for transfection efficiencies by dividing the relative light units by renilla activity. Each data point was the average of triplicate and one representative was shown.

### Primary hepatocytes isolation and adenovirus infection

Mice were anesthetized and livers were exposed surgically. Liver was first perfused with 25 ml of Solution I (9.5 g/l Hank’s balanced salt solution, 0.5 mmol/l EGTA, pH 7.2) and followed by 50 ml of Solution II (9.5 g/l Hank’s balanced salt solution, 0.14 g/l collagenase IV, and 40 mg/l trypsin inhibitor, pH 7.5). After perfusion, liver was chopped finely in a Petri dish and filtered through 45-μm pore mesh. Hepatocytes were then suspended in 50% percoll (Sigma), collected by centrifugation, and seeded onto collagen type 1 coated dishes in William E medium (Sigma). After 2 hr of incubation, medium was exchanged with DMEM with 10% FBS. At second day hepatocytes were infected with viral supernatant for 2 hr and collected at 24 hr post-infection followed by RNA isolation or western blot.

### Analysis of serum bile acid (BA), BA pool size, and fecal BA extraction

Serum total BA was determined by BA colorimetric assay BQ kit (Thermo Fisher Scientific, #BQ092A-EALD) according to the manufactures’ protocols. To determine bile acid pool size, fresh mice tissues including gallbladder, liver, and entire small intestine were minced and extracted in 75% ethanol at 50 °Cfor 2 hr. The extract was then centrifuged, diluted with 75% ethanol, and further diluted with 25% phosphate-buffered saline before the enzymatic reaction. The pool size was expressed as micromoles of bile acid/100 g of body weight. To determine fecal bile acid excretion, the feces from individually housed mouse over a 72 hr period were collected, weighed, dried, and extracted in 75% ethanol. The extract was diluted with 25% phosphate-buffered saline and subjected to bile acid measurement. The daily fecal output (grams/day/100 g of body weight) and fecal bile acid content (micromoles/g) were used to calculate the rate of bile acid excretion (micromoles/day/100 g of body weight).

### Serum Bile Acid Composition Determination

Bile acid standards were purchased from Steraloids (Newport, RI). Deuterated isotopes used as analytical standards were from C/D/N Isotopes, Inc. (Pointe-Claire, Québec, Canada). All chemicals and solvents were of highest grade. Methanol, ethyl-acetate, hexane, isooctane, 1-chlorobutane, and isoamyl alcohol were obtained from VWR (Montréal, Québec, Canada). Other reagents were purchased from Sigma Aldrich Co (Oakville, Ontario, Canada). Solide phase extraction (SPE) columns were from Phenomenex (Torrance, CA). Unconjugated, taurine-, glycine- and sulfate-conjugated bile acid levels were measured through liquid chromatography/tandem mass spectrometry (LC-MS/MS). Solid-phase extraction (SPE) was initiated by adding 2 mL of a 0.1% (w/v) formic acid solution and 30 μL of internal standards (i.e deuterated bile acids) to 200 μL of serum. The same treatment was applied to analytical standards which were diluted (1:1) with 200 μL of adsorbed serum, and subsequently used to generate calibration equations. SPE columns were conditioned with 1 mL MeOH and 2 mL of 0.1% formic acid. Columns were successively washed with 2 mL of H_2_O and 2 mL of H_2_O:MeOH (80:20) containing 0.1% formic acid under negative pressure. Bile acids were eluted with 2 mL of MeOH. Eluates were completely evaporated at 45 °C under N_2_ and reconstituted in 100 μL of H_2_O:MeOH (50:50) containing 5 mM ammonium acetate and 0.01% formic acid. Fifteen μL of sample or calibration standards were then injected into the LC-MS/MS system. A single LC method was used for the separation of the free, taurine, glycine and sulfate conjugates of bile acids. The chromatographic system consisted of an Alliance 2690 Separations Module (Waters, Milford, MA). Analytes were separated using a 50** × **3 mm Synergi Hydro-RP column (2.5-μm particles) (Phenomenex, Torrance, CA). The chromatographic conditions used were: 5 mM ammonium acetate-0.01% formic acid in water (solvent A), 5 mM ammonium acetate-0.01% formic acid in MeOH (solvent B), and acetonitrile (solvent C) at a flow rate of 800 μL/min. The chromatographic program was as follows: (i) initial conditions: 40% A: 55% B: 5% C for 4 min; (ii) a linear gradient to 80% B was applied over the next 8 min; (iii) the column was flushed with 90% B for the next 2 min; and (iv) re-equilibration to the initial conditions over the next 4 min. All analytes were quantified by tandem mass spectrometry (MS/MS) using an API4000 LC/MS/MS instrument (Applied Biosystems, Concord, ON, Canada). The analytical run required only 15 min, and the lower limit of detection varied from 0.5 (TUDCA) to 6.5 nM (GCDCA).

### Statistical Analysis

Data are expressed as mean ± SD. Statistical analyses were carried out using one-way ANOVA, followed by student’s t test; p < 0.05 was considered significant.

## Additional Information

**Accession Codes**: GEO accession number GSE67972 for RNA-seq analysis.

**How to cite this article**: Zhang, Y. *et al*. Bcl2 is a critical regulator of bile acid homeostasis by dictating Shp and lncRNA H19 function. *Sci. Rep.* **6**, 20559; doi: 10.1038/srep20559 (2016).

## Supplementary Material

Supplementary Information
