# Supplementary material for: Bcl2 is a critical regulator of bile acid homeostasis by dictating Shp and lncRNA H19 function
Source: Sci Rep. 2016 Feb 3;6:20559. doi: 10.1038/srep20559 (PMC4738356; doi:10.1038/srep20559)
Supplement: Supplementary Information [file srep20559-s1.doc]

**Bcl2 is a critical regulator of bile acid homeostasis by dictating Shp and lncRNA H19 function**

Authors: Yuxia Zhang, Chune Liu, Olivier Barbier, Rana Smalling, Hiroyuki Tsuchiya, Sangmin Lee, Don Delker, An Zou, Curt H. Hagedorn, Li Wang

**Supplemental Figures**

**
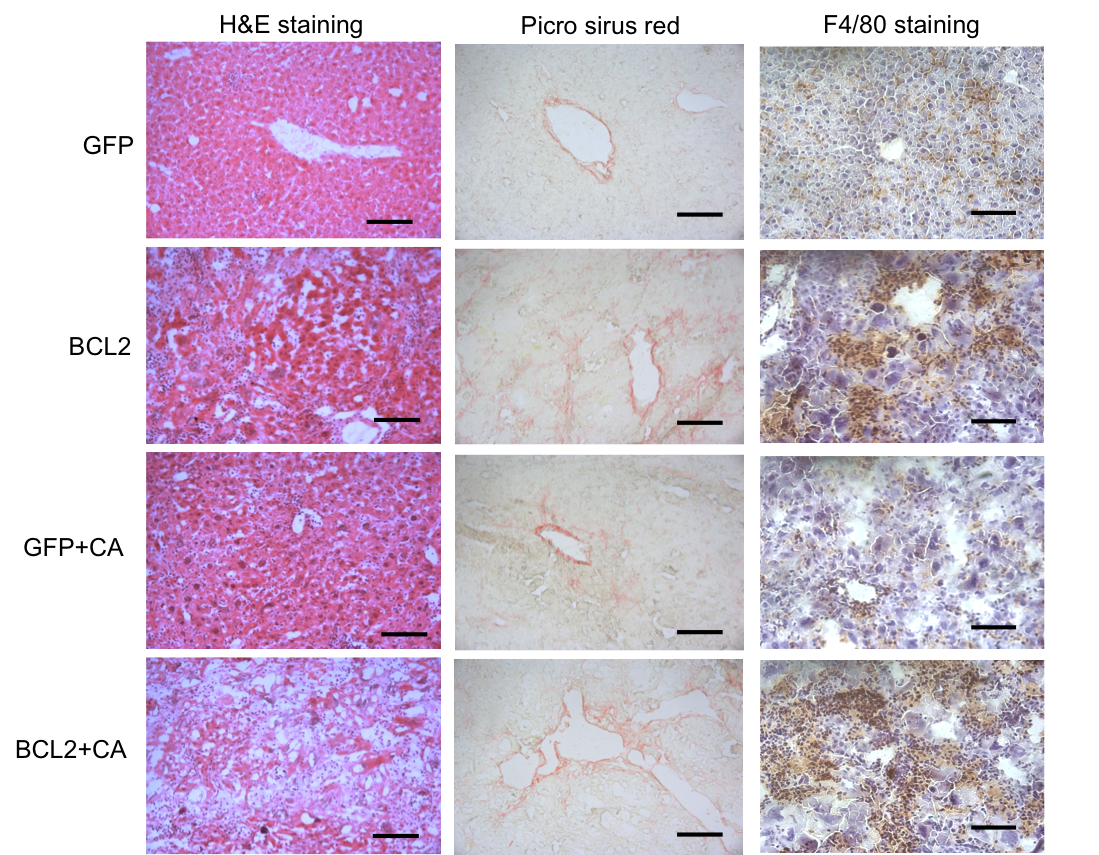
**

**Supp Figure 1.** Overexpression of Bcl2 induces an in vivo inflammatory response and fibrosis in liver. Bcl2 was expressed in C57BL6J mice by adenovirus mediated gene delivery for two weeks. A second group of mice were feed 1% cholic acid (CA) for 7 days one week after administration of adenovirus. H&E staining, picrosirius red staining of collagen, and immunohistochemical staining with the macrophage marker F4/80 in liver sections are shown as presented in the Fig. 2A. N=5 mice/group.


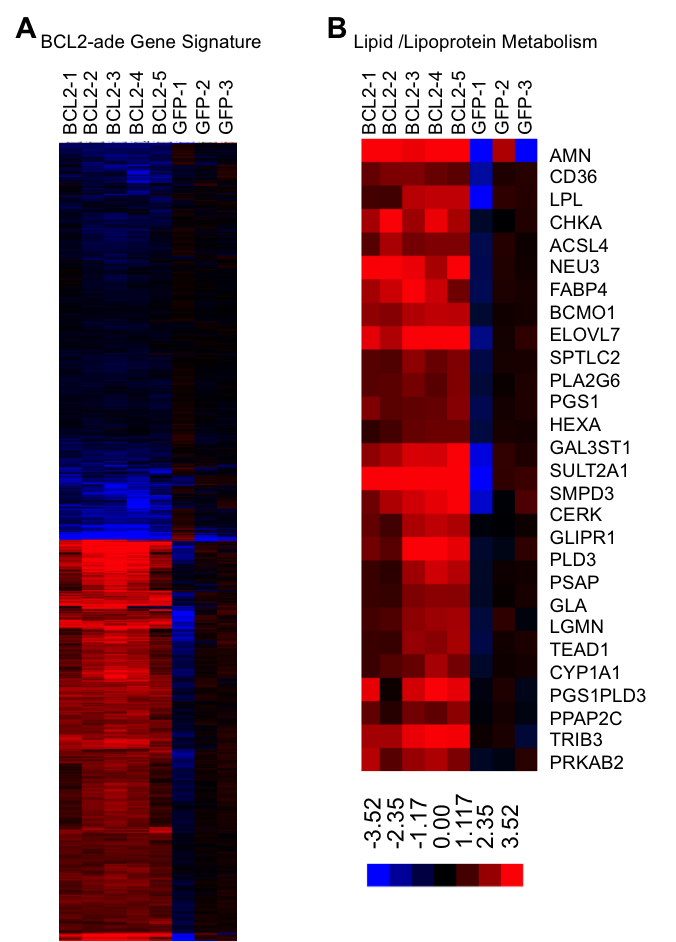


**Supp Figure 2.** RNA-seq reveals aberrant gene expression caused by Bcl2 overexpressing. (A) Unsupervised hierarchical clustering of the log-transformed gene expression reads for each sample showing a visual Bcl2 overexpression transcriptome signature of 1091 upregulated and 1073 downregulated genes. (B) Heat map showing differentially expressed genes (DEG) involved in lipid/lipoprotein metabolism.


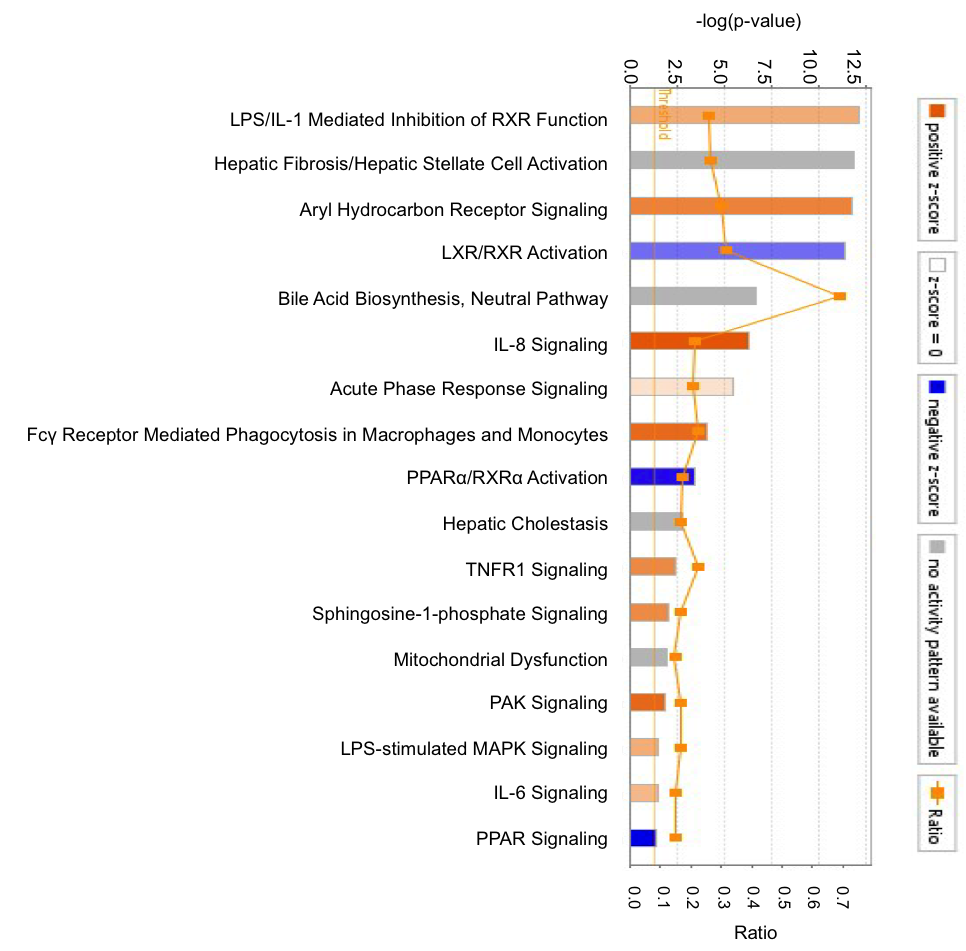


**Supp Figure 3.** Ingenuity pathway analysis showing top most relevant canonical pathways enriched in BCL2 liver. Z–score intensity reflects activation or de-activation of the pathway. All are significant with p<0.05.


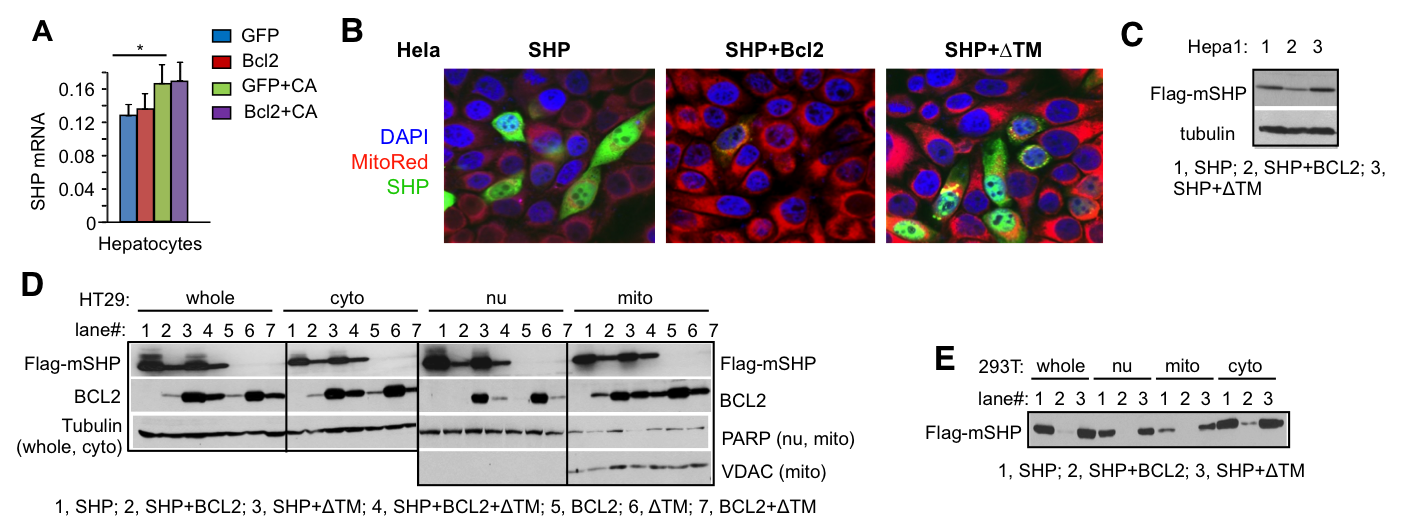


**Supp Figure 4.** Bcl2 destabilizes SHP protein in various cells.

(A) qPCR analysis of Shp mRNA in mouse primary hepatocyte subjected to adenovirus Bcl2 or GFP, alone or in combination with 100 μM cholic acid (CA) treatment for 24 hours. Data are represented as mean ± s.d. **P* < 0.01 *vs.* GFP.

(B) Micrographs of DAPI staining (blue), MitoTracker staining (red), and GFP-SHP expression (green) in Hela cells transfected with GFP-SHP plasmid, alone or in combination with wide-type Bcl2 or mutant Bcl2 lacking the TM domain (Bcl2ΔTM). Magnification x 20.

(C) Western blot of Flag-SHP protein in Hepa1 cells overexpressed with Flag-Shp, Bcl2 or Bcl2ΔTM.

(D-E) Western blot of Flag-Shp and BCL2 in sub-cellular fractionations from HT29 cells (d) and HEK293T cells (e) transfected with expression plasmids for Flag-mouse SHP, Bcl2, and Bcl2ΔTM, alone or in combination.


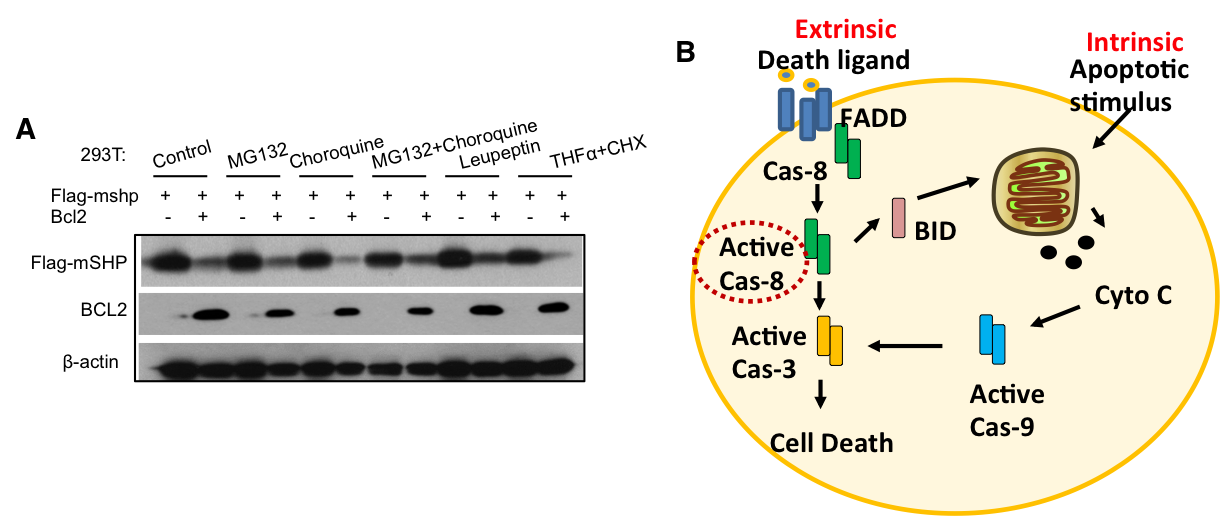


**Supp Figure 5.** (A) Western blot of Flag-Shp and BCL2 in HEK 293T cells transfected with expression plasmids Flag-Shp and Bcl2, alone or in combination. The cells were treated with for 6 hr with MG132 5 μM, choroquine 200 μM, leupeptin 100 μg/ml, TNFα 50 ng/ml + CHX 50 μM. (B) Schematic of extrinsic and intrinsic pathways leading to caspases activation in mammalian cells.


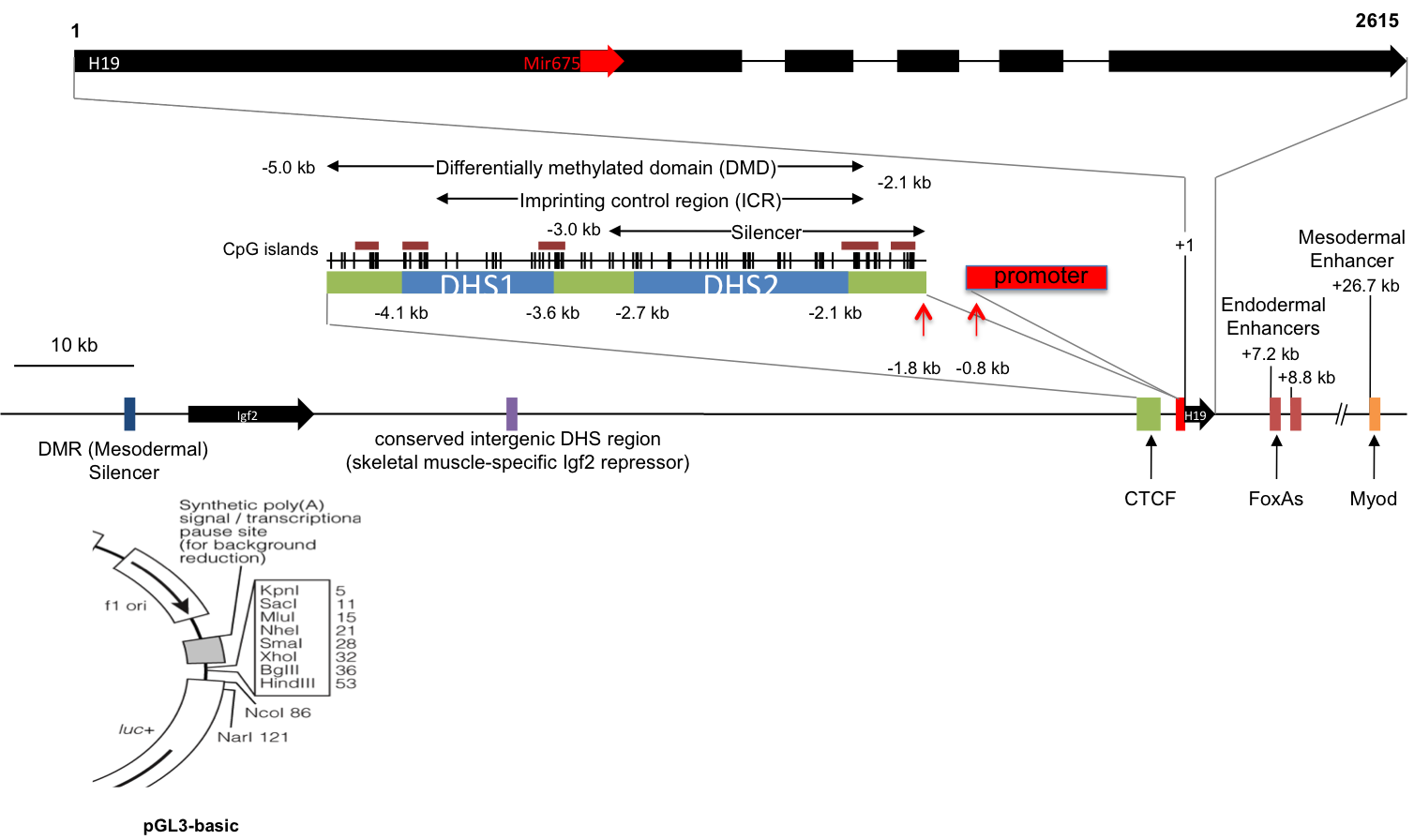


**Supp Figure 6.**  Schematic of mouse H19 gene structure showing H19 promoter. A 0.9 kb minimal promoter of H19 is constructed into a luciferase reporter.


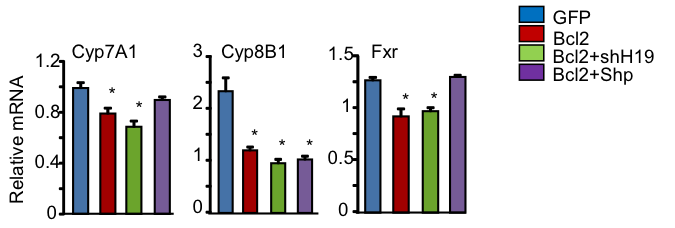


**Supp Figure 7.** qPCR analysis of hepatic gene expression in GFP, Bcl2, Bcl2+shH19 and bcl2+Shp mice. The mice were the same as in the main Fig. 5.
